# Application of a New Probabilistic Model for Mining Implicit Associated Cancer Genes from OMIM and Medline

**Published:** 2007-02-25

**Authors:** Shanfeng Zhu, Yasushi Okuno, Gozoh Tsujimoto, Hiroshi Mamitsuka

**Affiliations:** 1 Bioinformatics Center, Institute for Chemical Research, Kyoto University; 2 Graduate School of Pharmaceutical Sciences, Kyoto University

**Keywords:** Cancer genetics, Cancer gene discovery, Machine learning, Text mining, Probabilistic model

## Abstract

An important issue in current medical science research is to find the genes that are strongly related to an inherited disease. A particular focus is placed on cancer-gene relations, since some types of cancers are inherited. As biomedical databases have grown speedily in recent years, an informatics approach to predict such relations from currently available databases should be developed. Our objective is to find implicit associated cancer-genes from biomedical databases including the literature database. Co-occurrence of biological entities has been shown to be a popular and efficient technique in biomedical text mining. We have applied a new probabilistic model, called mixture aspect model (MAM) [48], to combine different types of co-occurrences of genes and cancer derived from Medline and OMIM (Online Mendelian Inheritance in Man). We trained the probability parameters of MAM using a learning method based on an EM (Expectation and Maximization) algorithm. We examined the performance of MAM by predicting associated cancer gene pairs. Through cross-validation, prediction accuracy was shown to be improved by adding gene-gene co-occurrences from Medline to cancer-gene cooccurrences in OMIM. Further experiments showed that MAM found new cancer-gene relations which are unknown in the literature. Supplementary information can be found at http://www.bic.kyotou.ac.jp/pathway/zhusf/CancerInformatics/Supplemental2006.html

## Introduction

Cancer is attributed to complex interactions of multiple factors, such as inheritance, gene mutation and environment. It is characterized by genetic alteration such as DNA amplification, deletion, translocation and point mutation, as well as distortion in gene expression [25]. Most known cancer-causing genes, oncogenes and tumor suppressor genes, have the crucial function of regulating cell proliferation, differentiation and death for cancer genesis and progression. New cancer therapy could target the proteins encoded by these genes to kill cancer cells or inhibit the propagation of them. Some other genes are highly expressed in cancer cells than normal cells, which could be utilized for early detection of oncogenesis [16]. Thus, the discovery of the cancer associated genes is extremely helpful for the understanding of tumor pathogenesis, and potential diagnosis and treatment of the cancer.

Linkage studies were first successfully used to find some cancer-susceptibility genes with high penetrance, such as *BRCA1* and *BRCA2* in breast cancer [6]. It examines the genotypes and phenotypes of parents and offspring in cancer families to locate the susceptibility genes, which will be further assessed and screened for validation. However, it lacks the power to detect multiple susceptibility alleles with moderate risks. Genetic association studies [7] alleviate this problem by comparing the genotype distribution between diseased individuals and non-diseased individuals for finding allelic variants that predispose to cancer. Because of the existence of linkage disequilibrium, genotype variants within a region can be captured by a subset of single-nucleotide polymorphisms (SNPs) [40]. Then the association candidate gene or genomic region with cancer could be examined by a tagging-SNP approach. With the increasing accumulation of SNPs data in genomic databases, such as the HapMap project [41], selecting a set of tagging SNPs that covers all common genetic variants in whole genome becomes possible [37].

To increase the success rate, the candidate genes could be selected for carrying out association studies. For example, with the complete sequencing of whole human genome, given a known cancer associated gene, we can find some possible homologous susceptibility-genes that have similar sequences by using sequence alignment programs, such as BLAST [1] and FASTA[35], or similar structures in the encoded protein. Furthermore, due to the rapid development of bioinformatics, more and more high throughput genomic data such as genomics, transcriptomics, proteomics and metabonomics data, as well as novel algorithms for effectively and efficiently integrating and analyzing these data, could be utilized to improve the selection of candidate genes. The genetic alteration in cancer cells could be identified by molecular cytogenetic techniques and comparative genomic hybridization (CGH) approaches [23, 11]. Subsequent gene expression pattern changes could be captured (or dissected) by analyzing the microarray gene expression profile, and digital expression pattern data such as expression sequence tags (ESTs) [4] and serial analysis of gene expression (SAGE) [42]. Proteomic and metabolic data can also provide valuable biological insights on cancer gene discovery.

By contrast, in this work, we attempt to mine implicit associated cancer genes that do not appear in the literature by applying a new probabilistic model, mixture aspect model (MAM) [48] on cancer gene co-occurrence data in OMIM and Medline. Online Mendelian Inheritance in Man (OMIM), a comprehensive human curated knowledgebase of human genes and genetic disorders, was first created by Victor McKusick at Johns Hopkins University, and now updated by him and other scientists [29, 17]. Until December 2005, it consists of more than 16,000 records, which can be divided into several categories based on genes, phenotypes or both. There are around 2,200 entries including both disease phenotype description and associated genes. Bajdik et al [2] wrote a software tool CGMIM to extract these entries to identify genetically-associated cancers and candidate genes by mapping those diseases into 21 type of cancers. Using this software, we can obtain two types of co-occurrence datasets: cancer gene and cancer-cancer co-occurrence datasets. MAM was proposed by us to mine implicit” chemical compound-gene” relations by integrating three types of co-occurrence datasets in the literature, i.e. gene-gene, compound-compound, and compound-gene. MAM was extended from a classical probabilistic model, aspect model (AM), which has been successfully applied in natural language processing, information retrieval, and collaborative filtering in E-commerce [19, 20]. The advantage of MAM, comparing with AM, is that MAM can handle different type of co-occurrence data, keeping the same time and space efficiency as those of AM. Thus, we can say AM is a special case, handling only one co-occurrence dataset, of MAM. We emphasize that this extension of AM to MAM is significant in the situation where we can use a lot of different types of co-occurrence datasets.

In addition to applying MAM on existing cancer-gene and cancer-cancer co-occurrence datasets from OMIM, we further incorporated gene-gene co-occurrences from a different data source, Medline [45], which can capture biological relationships among co-occurred genes. We first examined the performance of our model by cross-validation and found that combining all three types of co-occurrence datasets achieves the best result. This result indicates that MAM would be especially effective to predict an unknown gene, which is implicitly associated with some cancer, with a high accuracy. We then trained our model using all obtained co-occurrence datasets and predicted the likelihoods of unknown cancer-gene pairs, which are expected to be strongly related. We finally focused on unknown genes which are specific to each type of cancer and ranked them for each cancer, according to the likelihoods predicted by our trained model. The top 20 of these genes for each cancer are given as an online supplement material for cancer biologists’ reference, and we analyzed some of these genes from biological, medical and genetic viewpoints.

## Related Work

Genetic alteration of chromosomal aberrations and rearrangement, especially structural chromosome aberrations, could be discerned by using cytogenetic and molecular genetics techniques, such as G banding, fluorescence in situ hybridization (FISH) and spectral karyotyping (SKY) [38]. In contrast to above techniques, Comparative Genomic Hybridization (CGH) [23, 11] can scan entire genome in a single step to identify segmental DNA copy number changes by taking advantage of the complete sequencing of human genome project. Although FISH, SKY and CGH techniques have already been widely used and made significant impacts on cancer research, they could only achieve limited resolution of 5–20Mb in genomic DNA alteration identification. By incorporating latest microarray techniques, array-based CGH such as bacterial artificial chromosome (BAC) array CGH, cDNA array CGH and oligonucleotide array CGH, can achieve much higher resolution for discerning genomic DNA alteration [32, 33, 28]. Another high resolution technique digital karyotyping is based on enumerating the sequence tags to quantitatively measure DNA copy number change [44].

After the identification of amplified or deleted chromosomal regions, bioinformatics approaches can facilitate the discovery of cancer associated genes by analyzing the high-throughput biological data. Many studies have been carried out to analyze microarray gene expression data to find cancer related genes, which assumes that the expression level of one gene could be reflected by the abundance of corresponding mRNA. The most popular technique is to find differential expressed genes with high fold change between normal and tumor cells. For example, novel gastric cancer-related genes, specifically, such as potential marker CDC20 and MT2A, were discovered using a cDNA microarray [24]. Unlike microarray technology, digital expression profiling using expressed sequence tags (ESTs) or serial analysis of gene expression (SAGE) can be also used to identify cancer associated genes [4, 42]. In digital expression profiling, we assume that the expression level of one gene is proportional to the relative frequency of corresponding sequence tag in cDNA library data. Recently, Shen and his colleagues identified breast cancer related genes by analyzing differential gene expression between healthy and tumor breast tissue in EST and SAGE high throughput data [39]. After combining multiple analyses, they found six interesting genes related to breast cancer, with four down-regulated genes, ANXA1, CAV1, KRT5 and NMP7 and two up-regulated genes, ERBB2 and G1P3.

Although many studies analyzed high-throughput biological data to identify cancer associated genes, there are very few work that made use of literature mining. Mining biomedical text is attracting a great deal of interest because it can acquire accumulated biological and medical information and facilitate further knowledge discovery [47]. Some researchers already discovered disease gene candidates by text mining. For example, Freudenberg et al clustered diseases according to their phenotypic similarity and characterized genes with related GO function terms [13]. Potential disease genes from the human genome are then scored by their functional similarity to known disease genes in the same cluster of query disease. Perez-Iratxeta et al [30] used the fuzzy set theory to analyze the relationships between co-occurred MeSH terms in different categories, as well as the co-occurrence of a MeSH term and a GO (Gene Ontology) term in Medline records. Furthermore, they scored the implicit associations between symptoms of diseases and GO terms by fuzzy relations. In this work, we focus on mining the relationship of genetically-associated cancers and candidate genes, which can be obtained from the OMIM text database.

Most of text mining studies made use of co-occurrence techniques to discover possible biological relationships among different entities. This technique is based on the following hypothesis: if biological entity A co-occurs with biological entity B in the same biomedical document (eg a Medline record), A and B should be biologically related with high probability. This hypothesis was experimentally testified by many researchers [22, 8]. Here we also employ this method to obtain cancer-gene and cancer-cancer pairs by using a public available software CGMIM, which mines the description section of OMIM record. Since OMIM is a human curated database, the accuracy of our dataset is high. Furthermore, we incorporate gene-gene co-occurrence pairs from Medline. Although these gene-gene pairs are derived from a different source other than OMIM, we assume that co-occurred gene pairs in Medline should have much higher probability of associating with the same cancer than randomly generated gene pairs, which may help improve the prediction of cancer associated genes. This assumption is verified in our experiment (See the Data section for details).

## Method

### Notations

We use the same set of notations throughout the paper. A variable is denoted by a capitalized letter, and its value by corresponding lowercase letter. To explore the co-occurrence of a cancer and a gene in literature, let *G* be an observable random variable taking values *g*_1_, *g*_2_, ..., *g_S_*, each of which stands for a specific gene, and let *C* be an observable random variable taking value *c*_1_ *c*_2_,..., *c_T_*, each of which stands for a specific type of cancer. Similarly, let Z be a discrete valued latent variable taking on values *z*_1_ ..., *z_H_*, each of which corresponds to a latent cluster, where *H* is the number of clusters. Let θ be a set of parameters for the model to be optimized in the learning process, and let π be a mixture parameter (ie weight) of a component of our model that the users can specify. Let *D* be a set of all examples.

### Mixture Aspect Model for Predicting Cancer-Gene Co-occurrences

Aspect model (AM) was proposed by Hofmann for tackling problems in natural language processing [19, 20]. With latent clusters *z_h_*(*h* = 1, ..., *H*), AM gives the log-likelihood for a co-occurrence of (*c_i_*, *g_j_*) in the following form:

$$\text{log}p(c_{i},g_{j})=\text{log}\sum_{h}\;p(c_{i}\;|\;z_{h})\;p(g_{j}\;|\;z_{h})\;p(z_{h}).$$

Thus the log-likelihood for *D* by this model is given as follows:

$$\text{log}p(D)=\sum_{i,j}N_{i,j}\;\text{log}p(c_{i,}g_{j}),$$

where *N_i_*_,_ *_j_* is the number of co-occurrences of (*c_i_*, *g_j_*).

The objective of this work is to integrate different types of co-occurrence datasets, to identify cancer-associated genes with high accuracy. We used Mixture of Aspect Model (MAM), which was extended from AM by us in our previous work, to efficiently integrate different types of co-occurrence datasets. MAM has a general framework, and in this paper, we explain MAM briefly. Interested readers should refer to our previous paper [48], where the details of MAM are described. We denote the model built from *k* types of co-occurrence datasets as *k*MAM. For example, two types of co-occurrence datasets can be integrated by 2MAM. In this work, we have three types of co-occurrence datasets: cancer-gene from OMIM, cancer-cancer from OMIM, and gene-gene from Medline. Thus, we finally used 3MAM.

Here we focus on 3MAM which integrates all the three types of co-occurrence datasets. The models for other kinds of combinations among co-occurrence datasets could be derived similarly.

The log-likelihood for all data *D* can be given by 3MAM as follows:

$$\begin{array}{l} \text{log}p(D)=\pi_{CG}\sum_{i,j}\frac{N_{i,j}}{N_{CG}}\text{log}\sum_{h}p(c_{i}\;|\;z_{h})\;p(g_{j}\;|\;z_{h})\;p(z_{h})\\ \;\;\;\;\;\;\;\;\;\;\;\;\;\;\;\;\;\;\;\;\;+\pi_{GG}\sum_{j,j^{\prime }}\frac{M_{j,j^{\prime }}}{N_{GG}}\text{log}\sum_{h}p(g_{j}\;|\;z_{h})\;p(g_{j^{\prime }}\;|\;z_{h})\;p(z_{h})\\ \;\;\;\;\;\;\;\;\;\;\;\;\;\;\;\;\;\;\;\;\;+\pi_{CC}\sum_{i,i^{\prime }}\frac{L_{i,i^{\prime }}}{N_{CC}}\text{log}\sum_{h}p(c_{i}\;|\;z_{h})\;p(c_{i^{\prime }}\;|\;z_{h})\;p(z_{h}). \end{array}$$

In the above equation, π*_CG_* + π*_GG_* + π*_CC_* = 1, *N_CC_* = ∑*_i,i′_L_i,i′_*, and *L_i,i′_* is the number of (c*_i_*,c*_i′_*) pairs.

### Estimating Probability Parameters

Given training data *D* and the number of clusters *H*, a popular criterion for estimating the probabilities of a probabilistic model is the maximum likelihood (ML). Parameters are estimated to maximize the log-likelihood of data D:

$$\theta^{ML}=\text{arg}\underset{\theta }{\text{max}}\text{log}p(D;\theta ).$$

The most popular approach for obtaining an ML estimator of a probabilistic model is a time-efficient general scheme called the EM (Expectation-Maximization) algorithm [10] that provides a local maximum. In general, the EM algorithm starts with a random set of initial parameter values and iterates both the expectation step (E-step) and the maximization step (M-step) alternately until a certain convergence criterion is satisfied.

### Aspect Model

We begin to explain the EM algorithm for AM for only one type of co-occurrence dataset, i.e. cancer gene pairs. The log-likelihood for *D* is given in Section 3.2, and the E- and M-steps can be given as follows:

E-step:

$$p(z_{h}\;|\;c_{i},g_{j})=\frac{p(c_{i}\;|\;z_{h})\;p(g_{j}\;|\;z_{h})\;p(z_{h})}{\sum_{h^{\prime }}p(c_{i}\;|\;z_{h^{\prime }})\;p(g_{j}\;|\;z_{h^{\prime }})\;p(z_{h^{\prime }})}.$$

M-step:

$$\begin{array}{l} \hat{p}(c_{i}\;|\;z_{h})\propto \sum_{j}N_{i,j}\cdot p(z_{h}\;|\;c_{i},g_{j})\\ \hat{p}(g_{j}\;z_{h})\propto \sum_{i,j}N_{i,j}\cdot p(z_{h}\;|\;c_{i},g_{j})\\ \;\;\;\;\;\hat{p}(z_{h})\propto \sum_{i,j}N_{i,j}\cdot p(z_{h}\;|\;c_{i},g_{j}) \end{array}$$

### Mixture Aspect Model

Now we show the EM algorithm for 3MAM which can use all the three types of co-occurrence data-sets: cancer-gene, gene-gene and cancer-cancer pairs. To maximize the log-likelihood described in Section 3.2, the E- and M-steps for 3MAM can be given as follows:

E-step:

$$\begin{array}{l} p(z_{h}\;|\;c_{i},g_{j})=\frac{p(c_{i}\;|\;z_{h})\;p(g_{j}\;|\;z_{h})\;p(z_{h})}{\sum_{h^{\prime }}p(c_{i}\;|\;z_{h^{\prime }})\;p(g_{j}\;|\;z_{h^{\prime }})\;p(z_{h^{\prime }})}\\ p(z_{h}\;|\;g_{j},g_{j^{\prime }})=\frac{p(g_{j}\;|\;z_{h})\;p(g_{j^{\prime }}\;|\;z_{h})\;p(z_{h})}{\sum_{h^{\prime }}p(g_{j}\;|\;z_{h^{\prime }})\;p(g_{j^{\prime }}\;|\;z_{h^{\prime }})\;p(z_{h^{\prime }})}\\ p(z_{h}\;|\;c_{i},c_{i^{\prime }})=\frac{p(c_{i}\;|\;z_{h})\;p(c_{i^{\prime }}\;|\;z_{h})\;p(z_{h})}{\sum_{h^{\prime }}p(c_{i}\;|\;z_{h^{\prime }})\;p(c_{i^{\prime }}\;|\;z_{h^{\prime }})\;p(z_{h^{\prime }})} \end{array}$$

M-step:

$$\begin{array}{l} \hat{p}(g_{j}\;|\;z_{h})\propto \pi_{cg}\;\sum_{i^{\prime }}\frac{N_{i,j}}{N_{CG}}\;p(z_{h}\;|\;c_{i},g_{j})\\ \;\;\;\;\;\;\;\;\;\;\;\;\;\;\;\;\;\;\;\;+\pi_{CC}\;\sum_{i^{\prime }}\frac{L_{i,i^{\prime }}}{N_{CC}}\;p(z_{h}\;|\;c_{i},c_{i^{\prime }})\\ \hat{p}(g_{j}\;|\;z_{h})\propto \pi_{CG}\;\sum_{i}\frac{N_{i,j}}{N_{CG}}\;p(z_{h}\;|\;c_{i},g_{j})\\ \;\;\;\;\;\;\;\;\;\;\;\;\;\;\;\;\;\;\;\;\;+\pi_{GG}\;\sum_{j^{\prime }}\frac{M_{j,j^{\prime }}}{N_{GG}}\;p(z_{h}\;|\;g_{j},g_{j^{\prime }})\\ \hat{p}(z_{c})\propto \pi_{CG}\;\sum_{i,j}\frac{N_{i,j}}{N_{CG}}\;p(z_{h}\;|\;c_{i},g_{j})\\ \;\;\;\;\;\;\;\;\;\;\;\;+\pi_{GG}\;\sum_{j^{\prime },j^{\prime \prime }}\frac{M_{j,j^{\prime \prime }}}{N_{GG}}\;p(z_{h}\;|\;g_{j^{\prime }},g_{j^{\prime \prime }})\\ \;\;\;\;\;\;\;\;\;\;\;\;\;\;+\pi_{CC}\;\sum_{i^{\prime },i^{\prime \prime }}\frac{L_{i^{\prime },i^{\prime \prime }}}{N_{CC}}\;p(z_{h}\;|\;c_{i^{\prime }},c_{i^{\prime \prime }}) \end{array}$$

### Parameter Settings in Our Experiments

We set the number of latent clusters, H, at 128 and used a uniform distribution for the weights (ie π) of both 2MAM and 3MAM in all cases. We iterated the EM algorithm until the improvement of the observed log-likelihoods between two successive iterations is less than 0.001.

## Data

### Cancer-Gene and Cancer-Cancer Co-occurrences

OMIM (Online Mendelian in Man) is a human-curated database, containing the comprehensive and authoritative information on human genes and genetic disorders. Our focus is placed on genes which are related with cancers, and we used a software tool CGMIM, which extracts the description section of OMIM records to obtain cancers and associated genes. The CGMIM builds a synonym list from International Classification of Disease for Oncology (ICD-O) [14]. The list maps genetic disorders into 21 different types of cancers, which are defined by the National Cancer Institute of Canada. They are bladder, brain, breast, cervix, colorectal, esophagus, kidney, larynx, leukemia, lung, lymphoma, melanoma, myeloma, oral, ovary, pancreas, prostate, stomach, testis, thyroid and body-of-uterus. We obtained the two types of co-occurrence datasets from the OMIM database downloaded in Oct 2005. Our datasets are altogether 2,017 genes associated to cancers, 3,743 cancer-gene pairs and 206 cancer-cancer pairs.

### Gene-Gene Co-occurrences

Since gene-gene co-occurrences are not available in OMIM, we obtained this kind of co-occurrences from the Medline database. We used Locuslink [34], ie a human curated database, to avoid errors that may occur in identifying gene names in Medline. The Locuslink has a list of links, each of which connects a Locus ID with a PubMed ID, meaning that we can see whether a gene (specified by a Locus ID) is in an abstract (specified by a PubMed ID) or not.

We used a file available at the following ftp site, and the file we used was generated at Dec 2004:

ftp://ftp.ncbi.nih.gov/refseq/LocusLink

From this list, we selected Medline records containing one or more human genes, focusing on “human” genes only. We then generated gene-gene co-occurrences from the selected Medline records. That is, if two genes are in a same Medline record, we can say that these two genes co-occur.

We found some Medline records have a large number of genes. For example, a record with PubMed ID 12477932 contains more than 9,000 human genes by showing all genes in a microarray experiment. Thus, we removed the record, each of which has more than 10 genes. We note that this is a normal procedure in dealing with Medline records. For example, Wilkinson et al also put this kind of restriction to filtering Medline records for finding communities of related genes [46].

Our focus is on cancer associated genes, and a gene-gene co-occurrence pair was removed unless both genes of the pair are in the 2,017 genes of our cancer-gene co-occurrence dataset. Finally we obtained 3,118 gene-gene pairs from Medline. Table 1 shows a summary of the data information.

### Preliminary Verification on Gene-Gene Co-occurrence Dataset

Focusing on genes in cancer-gene co-occurrence pairs from OMIM, we attempted to confirm that two genes in each gene-gene pair from Medline are associated to a same cancer with high probability. When both two genes in a gene-gene pair are associated with at least one same cancer, we call such a gene-gene pair a *positive pair,* and we computed the ratio of positive pairs to all gene-gene pairs, which we call the *positive ratio.*

We found that among total 3,118 gene-gene co-occurrence pairs, 1,804 (57.86%) are positive pairs. We then reduced the size of gene-gene pairs by the number of co-occurrences and checked the positive ratio. Table 2 summarizes the obtained results.

As shown in the table, with increasing the co-occurrence number of gene-gene pairs, the positive ratio increased. For example, when the number of co-occurrences is set at more than one, 490 (64.64%) out of 758 gene-gene pairs are positive pairs. Furthermore, as a baseline, we checked the positive ratio of randomly generated pairs. That is, we randomly generated 3,118 gene-gene pairs 1,000 times using our 2,017 cancer associated genes and checked the average positive ratio for them. The average positive ratio was only 26.65%, with minimum 24.05%, maximum 29.76% and standard deviation 0.0083, which is far less than those obtained by our gene-gene co-occurrence dataset. These results clearly indicate that the motivation of adding gene-gene co-occurrence data in Medline to the cancer-gene and cancer-cancer data from OMIM would be reasonable.

## Experimental Results

### Predictive Performance of Mixture Aspect Model

#### Evaluation Procedure

We evaluated the performance of MAM by cross-validation on predicting associated cancer-gene pairs. We examined four types of MAM (including AM). That is, we first built AM using only the cancer-gene co-occurrence dataset. We then tested two different 2MAM by adding cancer-cancer or gene-gene pairs the cancer-gene pairs, which correspond to 2MAM (CG+CC) or 2MAM (CG+GG), respectively. Finally 3MAM was examined by using all these three types of co-occurrence datasets.

To examine the effect of the training data size on the performance of our models, we checked three different data-size ratios of training to test datasets, 3:1, 1:1 and 1:3, in our cross-validation experiment. For example, in the 1:1 case, we randomly divided the original cancer-gene dataset into two subsets of roughly equal size, and then alternately selected one subset as a test set and the other as a training set. We carried out 50 rounds of the cross-validation to reduce the possible biases caused by random partitioning. In each round, to compare the performance different models, we kept the testing dataset unchanged while adding another type of co-occurrence dataset. In this way, we made predictions on the same test dataset. We note that AM cannot compute the likelihood for a cancer gene pair in the test dataset unless a gene of this pair appears in the training data. So we removed all the pairs which are not in the training data but in the test dataset. We then used all remaining pairs as positive test examples. Please note that this experimental setting advantageous to AM and not to MAM. Negative examples, which were used for evaluation only, were randomly generated to be included in neither the training dataset nor the positive test dataset. The size of negative test dataset was set as the same as that of positive test dataset.

#### Evaluation Measures

1. Area Under the ROC Curve (AUC)
  The performance of each probabilistic model is evaluated by the ability to discriminate positive examples from negative examples in test data of our cross-validation. We used AUC (Area Under the ROC curve) to evaluate the discriminative performance of a model. The AUC is computed from an ROC (Receiver Operator Characteristic) curve. The ROC curve is drawn by plotting “sensitivity” against “false positive rate”, using the ranked cancer-gene pairs. The sensitivity (or true positive rate) is the proportion of the number of correctly predicted positive examples to the total number of positive examples. The false positive rate is the proportion of the number of false positive examples to the total number of negative examples. More concretely, once we estimated the parameters of a probabilistic model from training data, we computed the likelihood of each cancer-gene pair in test data and ranked them according to their likelihoods. We then set a cut-off value to separate positive examples from negatives and computed the sensitivity and the false positive rate by changing the cut-off value from the highest likelihood to the lowest. We finally plotted all obtained values of the sensitivity and the false positive rate to draw an ROC curve.
  The AUC, a popular metric for measuring the performance of different models [5, 18], can be computed as the area under this ROC curve. We can see that the larger the AUC, the better the performance of the model. We further used the paired sample two-tailed *t-*test to statistically evaluate the performance difference between 3MAM and another model. Since we run crossvalidation 50 times, we have at least 100 values in each of the three different ratios, and so if the *t*-value is greater than 3.50 (2.36) then the difference is more than 99.9% (98%) statistically significant.
2. Log-likelihood Distribution on Positive Test All these four probabilistic models are trained in an unsupervised manner and the maximum likelihood setting, meaning that they are trained to provide the maximum likelihoods to given training data. In addition, conveniently enough, they have the same (common) set^1^ of parameters, ie *p*(*c_i_|z_h_*), *p*(*g_j_|z_h_*) and *p*(*z_h_*). Thus, we can compare the four models each other by the distribution of the likelihoods for positive test examples, given by each of the models. If a model provides positive examples with higher likelihoods than those of another, we can say that this model is better than the other.

#### Results

1. AUC
  Table 3 shows the AUC for each of the four models at different data settings and the t-value (in parenthesis) between the AUC of 3MAM and that of another model.
  This table clearly shows that 3MAM outperformed the other three models, and the second best model is 2MAM (CG+CC). We can easily see that, compared with AM, the 3MAM improved around 2 to 9% in the discriminative accuracy. Furthermore, the *t-*values showed that 3MAM outperformed all other models by a statistically significant factor in all cases. These results indicate that incorporating cancer-cancer and gene-gene pairs from diverse sources improved the predictive performance obtained by cancer-gene pairs only.
  In addition, we note the following two points on these results: First, interestingly, 2MAM (CG+GG) outperformed AM in 1:1 and especially 1:3 cases, but not 3:1 case. This is probably because gene-gene co-occurrence data comes from the different source, Medline, which can supplement original data, when it is scarce, and can achieve better performance. Second, since we have only 21 type of cancers and 2,017 genes, some putative negative test examples must be positive. This means that the performance of our model may be underestimated.
2. Log-likelihood Distribution on Positive Test When the probability parameter has a uniform distribution, a randomly generated cancer-gene pair has the following log-likelihood:
  $$\text{log}\left(\frac{1}{21}\times \frac{1}{2,017}\right)=-4.63$$
  In our unsupervised setting, the log-likelihood of a positive example should be larger than the above value. In other words, when positive (test) examples are given, a better trained probabilistic model would provide a larger number of examples whose log-likelihoods are larger than the above value.

**Figure 1:**
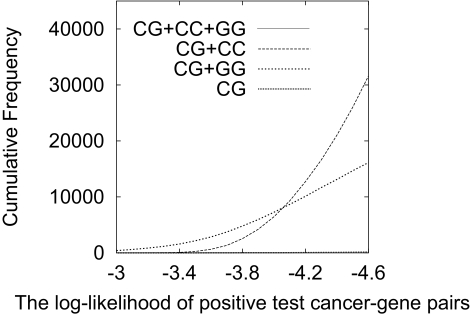
Cumulative number of positive examples with higher log-likelihoods.

Thus, given a cut-off value, we checked the number of positive test examples having log-likelihoods larger than the given cut-off value. Figure 1 shows the counted cumulative number of positive test pairs with higher likelihoods against a given cut-off value. This figure is drawn from the average over the 50 rounds of our cross-validation at the 3:1 ratio of training to test data. We found that 3MAM is clearly the best among the four models, always keeping the largest number of examples whose likelihoods higher than a given cut-off value. These results also confirmed the performance advantage of 3MAM over other models and showed adding cancer-cancer and cancer-gene datasets is effective. Another empirical finding in this analysis is that 2MAM (CG+GG) outperformed 2MAM (CG+CC) in the range of larger than −4, while 2MAM (CG+CC) outperformed 2MAM (CG+GG) in the range between −4.6 and −4.

### Mining and Analyzing Unknown Cancer Associated Genes

#### Mining New Cancer-Gene Co-occurrences

We trained 3MAM using all three types of co-occurrence data and tried to find new associated cancer gene pairs which are unknown in the current literature. The procedure is as follows: We first trained 3MAM using all the three types of co-occurrence data and then computed the log-likelihoods of all cancer-gene paris that are not in the current cancer-gene co-occurrence data. We repeated this procedure 100 times and ranked the new pairs according to the average log-likelihoods over 100 times. Table 4 shows the list of top 20 pairs with their log-likelihoods, and a more detailed list of top 1,000 pairs is given in Table 1 of the on-line supplementary information. The first, second, third and fourh columns of the on-line information show cancer names, HUGO IDs [43], genes and log-likelihoods, respectively.

As shown in Table 4, the top 20 list has some famous oncogenes such as TP53, BCL2 and TNF. This result implies that our prediction worked well, because these popular genes must be related with a lot of different types of cancers. So we can expect that these relations must exist, even if the cancer-gene co-occurrences in Table 4 are not in OMIM. In other words, we may say that these relations are easily expected. Thus in the next section, we focused on genes which are specific to some cancer but unknown and tried to analyze how the found genes are related with the corresponding cancer.

#### Mining New Genes Specific to Cancer

We computed the following score for all cancer-gene pairs by using the probability parameters of 3MAM, which was trained by using all three types of training data.

$$R(g_{j},c_{i})=\frac{p(g_{j}\;|\;c_{i})}{\sum_{i}p(g_{j}\;|\;c_{i})}$$

where

$$p(g_{j}\;|\;c_{i})=\frac{\sum_{h}p(c_{i}\;|\;z_{h})\;p(g_{j}\;|\;z_{h})p(z_{h})}{\sum_{j^{\prime },h^{\prime }}p(c_{i}\;|\;z_{h^{\prime }})\;p(g_{j^{\prime }}\;|\;z_{h^{\prime }})\;p(z_{h^{\prime }})}.$$

The *p*(*g_j_* | *c_i_*) is the conditional probability that given a cancer type *c_i_*, *g_j_* is related with the *c_i_*. Thus the score *R*(*g_j_*, *c_i_*) is the ratio that a gene *g_j_* is related with *c_i_*, comparing to all the other cancer types. That is, it is the probability over cancer types and shows to what extent gene *g_j_* is specific to cancer *c_i_*. Once we computed the score for each pair, we sorted the values for each cancer and selected the top 20 genes which are not in the cancer-gene pairs in the training data. Table 2 of the on-line supplementary information shows the list of top 20 genes of each cancer. The first, second, third and fourh columns of this file show cancer names, HUGO IDs, genes and parameter values, respectively.

These pairs are unknown pairs in OMIM and Medline, but our method suggested that each of them has a strong relationship between a cancer and a gene. In fact, we can see a biological relationship for each pair from the literature. Below we briefly describe the biological, medical and genetic relationships on each pair of the list, for only the top gene of seven cancers out of all 21 cancers, owing to the space limitations.

#### Brain:

The top is MMP17. According to Puente et al [36], they revealed that MMP17 is expressed mainly in the brain, leukocytes, colon, ovary and testis, using northern blot analysis of polyadenylated RNAs isolated from a variety of human tissues. This implies MMP17 can be related with brain cancer.

#### Breast:

The top is ZAP70, a member of the Syk tyrosine kinase family. Recently, Gatalica and Bing [15] pointed out that the loss of Syk tyrosine kinase expression characterises a subset of breast carcinomas. This implies a relationship between ZAP70 and breast cancer.

#### Colorectal:

The top is CYP1A1. Hou et al [21] recently reported the relationship between the CYP1A1 polymorphism and the risk for colorectal adenoma. Their summary is that the joint carriage of CYP1A1 and NQO1 polymorphisms, particularly in smokers, was related to colorectal adenoma risk, with a propensity for formation of multiple lesions. This would be an evidence for the relationship between CYP1A1 and colorectal cancer. The second is MAD2. The expression profile of MAD2 in colorectal cancer was investigated by Li et al [26]. Their result shows that the defect of spindle checkpoint gene MAD2 is involved mainly in colorectal carcinogenesis. So this clearly indicates the relationship between MAD2 and colorectal cancer.

#### Lymphoma:

The top is LMO1. In the recent study of leukemogenesis, Lin et al [27] found that almost 60% of transgenic mice that overexpressed both OLIG2 and LMO1 developed pre-T LBL with large thymic tumor masses. This reveals the association between LMO1 and lymphoma cancer.

#### Pancreas:

The top is NR5A2. NR5A2, a member of a nuclear receptor subfamily, is a liver recepter homolog1 (LRH-1). Fayard et al [12] showed that LRH-1 is abundantly expressed in pancreas. Furthermore, their in situ hybridization and gene expression studies demonstrated that both LRH and carboxyl ester lipase (CEL) are co-expressed and confined to the exocrine pancreas.

#### Prostate:

The top is KLK10, ie kallikrein 10. Bharaj et al [3] showed the association between single nucleotide polymorphisms in the human KLK10 and prostate cancer. Petraki et al [31] studied the localization of human KLK10 in benigh and malignant prostatic tissues and the correlation between the expression of KLK10 and prostate cancer (PC) prognosis. They pointed out that kallikreins may function as tumor suppressors or are down-regulated during cancer progression. These results imply the relationship between KLK10 and prostate cancer.

#### Testis:

GAGEB1 is the top. Chen et al [9] isolated GAGEB1 by differential display PCR. They found that GAGEB1 expression was restricted to testes and placenta on human multiple tissue Northern blots. This shows some relationship GAGEB 1 and testis cancer.

## Concluding Remarks

We have applied a new probabilistic model MAM, which was proposed by us in our research on mining implicit chemical compound-gene relationship, to the problem of finding new cancer associated genes from OMIM and Medline. MAM can integrate different types of co-occurrence datasets effectively, and we found that MAM performed very well even when co-occurrence datasets are gathered from heterogeneous sources.

In this work, we used a uniform distribution for the component weights (π) of our mixture model to allow users additional control. Interesting future work would adjust the weights to achieve the maximum predictive performance. On the other hand, the gene-gene co-occurrence data can come from a different source other than Medline. Since microarray expression data can reveal the biological relationship of genes, it would be very interesting to integrate gene-gene co-occurrence data from microarray expressions.

## Figures and Tables

**Table 1:** The size of co-occurrence datasets.

Item	Size
gene type	2,017
gene-gene	3,118
cancer type	21
cancer-cancer	206
cancer-gene	3,743

**Table 2:** The ratio of positive pairs in gene-gene co-occurrence dataset.

# co-occurrences	-(random)	> = 1	>1	> 2	> 3	> 4	> 5	> 6
Dataset size	3,118	3,118	758	379	276	152	122	99
Positive ratio (%)	26.65	57.86	64.64	68.34	69.91	70.2	72.13	76.77

**Table 3:** AUCs and *t*-values (in parenthesis) obtained by 50 rounds of cross-validation on cancer-gene pairs.

Model	**Ratio of training to test data**		
	3:1	1:1	1:3
3MAM (CG+CC+GG)	**76.1**	**74.6**	**73.2**
2MAM (CG+CC)	75.8 **(2.56)**	**74.2** **(2.44)**	**71.8** **(12.9)**
2MAM (CG+GG)	73.9 **(17.2)**	**71.4** **(22.5)**	68.3 **(38.0)**
AM (CG)	**74.1** **(14.7)**	70.5 **(26.3)**	64.9 **(55.1)**

**Table 4:** 20 Cancer-gene pairs with highest log-likelihoods that are not in our training dataset.

Cancer Type	Gene Name	Log-likelihood
OVARY	TP53	− 3.078
COLORECTAL	BCL2	− 3.085
STOMACH	TP53	− 3.113
LEUKEMIA	CDKN1A	− 3.176
LYMPHOMA	BAX	− 3.191
PANCREAS	TP53	− 3.199
BREAST	NFKB1	− 3.222
THYROID	TP53	− 3.234
LYMPHOMA	TNF	− 3.235
LUNG	BCL2	− 3.244
BREAST	BCL2	− 3.266
KIDNEY	TP53	− 3.269
BREAST	TNF	− 3.293
LEUKEMIA	TNF	− 3.300
COLORECTAL	TNF	− 3.312
LYMPHOMA NF	NFKB1	− 3.316
LUNG	TNF	− 3.323
COLORECTAL	CASP8	− 3.330
LEUKEMIA	NFKB1	− 3.336
BRAIN	BCL2	− 3.340

## Footnotes

1 We note that trained models have different parameter values because the training algorithms are different.
